# Finding fault types of BLDC motors within UAVs using machine learning techniques

**DOI:** 10.1016/j.heliyon.2024.e30251

**Published:** 2024-04-28

**Authors:** Dragos Alexandru Andrioaia, Vasile Gheorghita Gaitan

**Affiliations:** a "Vasile Alecsandri" University of Bacau, Bacau, 600115, Romania; b "Stefan cel Mare" University of Suceava, Suceava, 720229, Romania

**Keywords:** BLDC motor defects, UAV fault identification, PdM, Machine learning, UAV

## Abstract

Due to the potential of the Unmanned Aerial Vehicle (UAV), they began to be increasingly used in various fields such as: environment, leisure, health, military, transport, etc. Along with increasing battery storage capacity, the UAVs began to be propulsion by Brushless DC (BLDC) motors. Failure of BLDC motors can lead to loss of control, which can cause accidents. In these conditions, it is necessary to devise methods that can find the defects of the BLDC motors in the UAVs. In this article, the authors propose a novel method to predict BLDC motor defects using machine learning. To maximize the method results, the performance of three machine learning models, K-Nearest Neighbor (KNN), Support Vector Machine (SVM) and Bayesian Network (BN) in predicting the flaws of BLDC motors, have been compared.

## Nomenclature

**Abbreviations/Acronyms**
ANFIS: Adaptive Neuro Fuzzy Inference System
ANN: Artificial Neural Networks
BLDC: Brushless DC
BN: Bayesian Network
CNN: Convolutional Neural Network
DT: Decision Trees
FFT: Fast Fourier Transform
IoT: Internet of Things
KNN: K-Nearest Neighbor
PdM: Predictive Maintenance
PNN: Probabilistic Neural Network
PWM: Pulse-Width Modulation
RF: Random Forest
RMS: Root Mean Square
RUL: Remaining Useful Life
STFT: Short-Time Fourier Transform
SVM: Support Vector Machine
UAV: Unmanned Aerial Vehicle
WPT: Wavelet Packet Transform
WT: Wavelet Transform

**Variable/Parameter**
*ACC*: Accuracy
*C*: Penalty factor
*Cj*: Class number *j*
*C*_*P*_: Target value
*C1*: Healthy class
*C2*: Eccentric shaft class
*C3*: Chipped propeller class
*C4*: ESC fault (faulty FDD8896 MOSFET transistor) class
*d(x,x’)*: Distance between a test instance *x'* and each instance in the formation dataset *x*
*F1 Score*_*class j*_: F1 score for the class *j*
*F1score*_*Macro*_: Macro-averaged F1 score
*FN*: False negative rate
*FN*_*class j*_: False negative rate for the class *j*
*FP*: False positive rate
*FP*_*class j*_: False positive rate for class *j*
*I*: Electric current intensity of the BLDC motor
*k, m*: Total number of instances
*M Acc*: Acceleration magnitude of the BLDC motor
*P(C*_*j*_*)*: Class *C*_*j*_ probability
*P(X*_*i*_*│C*_*j*_*)*: Probability of *X*_*i*_ to be in *C*_*j*_
*PR*_*class j*_: Precision for the class *j*
*PR*_*Macro*_: Macro average precision
*Recall*_*class j*_: Sensitivity for the class *j*
*Recall*_*Macro*_: Macro average recall
*T*: Temperature
*TN*: True negative rate
*TP*: Truth positive rate
*TP*_*class j*_: Truth positive rate for the class *j*
*U*: Supply voltage of the BLDC motor driver
*w*: Vector weight
*x*: Instance of the training data set
*x’*: Test instance
*X*: Input vector
*X*_*i*_: Instance number *i*
*X*_max_: Maximum value of data after normalization
*X*_min_: Minimum value of data after normalization
*X*_*new*_: New value after normalization
*X*_*old*_: Old value before normalization
*ξ*_*i*_: Slack variable
*ψ*: Function to map the input data *X* to a higher-dimensional space
*(x*_*i*_*, y*_*i*_*)*: *i* paired instances, input output

## 1 Introduction

Unmanned Aerial Vehicles (UAVs) are frequently used in various fields for purposes such as: surveillance, leisure, transport, environmental monitoring, search and rescue, mapping, research of areas affected by disasters as well as for finding targets and fighting. Accidents related to UAVs have increased in proportion to their number [[1], [2], [3]].

Investment in UAVs research has considerably increased lately due to the fact that they can replace people in some dangerous situations [3].

Most UAVs use Brushless DC (BLDC) motors due to the advantages they have. BLDC motors have a high-power density, and the lack of brushes increases their reliability. A BLDC motor is a synchronous electric motor that has the windings placed on the stator, and its rotor has a series of permanent magnets. The rotor spins by changing the direction of the magnetic fields generated by the stator coils [[4], [5], [6], [7], [8], [9]].

The motor's driver converts the constant DC voltage into a trapezoidal Pulse-Width Modulation (PWM) signal. The motor's speed and direction of rotation can be changed by controlling the period of PWM pulses. The position of the rotor of BLDC motors can be read via Hall sensors [4,8,[10], [11], [12]].

The fact that BLDC motors run at high-speed causes failures. The failures commonly observed in UAVs motors include propeller failures, eccentric failures as well as other electrical failures. Malfunction of the UAVs that occurs during flight should be identified and diagnosed in a timely manner [2,5,13].

The physical impact to which a motor can be subjected from the outside may cause bending of the motor shaft, deformation of the rotor, or permanent magnets to detach from the rotor assembly. These situations may affect the balance of the rotor and cause the motor to operate with vibrations and noises, affecting the bearings and causing it to overheat [1].

The motor bearing may deteriorate, due to wear over time, especially when operating through a medium with impurities or when the rotor loses its balance. The appearance of incipient defects in the bearing does not lead to an immediate failure, but the increase in degradation over time will generate a critical failure [1,2,14,15].

Winding failures on the stator can occur with loss of insulation on the windings. Overload operation and loss of rotor balance can cause heating of the windings, and the undissipated heat will cause loss of insulation that leads to the combustion of the motor winding [1,8,16].

The propeller in solidarity with the motor rotor may deteriorate upon contact with an obstacle. If the edge of the blade deteriorates, the increased aerodynamic resistance of the propeller will increase the energy consumption and reduce the angular velocity. The decrease in angular velocity will affect the traction [[1], [2], [3],^17^,^18^].

For the reasons listed above, it is necessary to design methods that can identify the faults of BLDC motors in electrically powered UAVs. In this paper, the authors propose a method using machine learning to identify faults in BLDC motors from UAVs. To train the model, training data from the sensors of the experimental stand used to extract the degradation features of BLDC motors were used. The data set contains information taken from sensors (temperature, current voltage and acceleration) in which fault and fault-free operation of BLDC motors is captured. Compared to other research, the data set contains a generous number of parameters. The model was trained to identify four classes such as: healthy class, chipped propeller class, eccentric shaft class and ESC fault (faulty FDD8896 MOSFET transistor) class. The fault classes listed above are commonly encountered in electrically powered UAVs. To improve the results of the method, the performance of classifiers such as K-Nearest Neighbor (KNN), Support Vector Machine (SVM) and Bayesian Network(BN) in predicting the flaws of BLDC motors, was compared. The obtained results prove that the model can be implemented within a Predictive Maintenance (PdM) system of UAVs.

Early detection of UAV motors faults can improve operational safety and lead to a longer service life.

### 1.1 State of art

In the specialized literature, various methods which take into account signals from sensors, acceleration, electrical current, voltage and acoustics to detect failures of motors and propellers of UAVs, have been proposed [1].

O. Yaman, F. Yol and A. Altinors propose a method that allows the identification of BLDC motor faults in UAVs, which is based on the analysis of sound signals. Sound signals were collected from the BLDC motors of several types of UAVs (helicopter, duocopter, tricopter, and quadcopter models). Feature extraction was made on the collected sound signals using Mel-frequency Cepstral Coefficients (MFCC) method and SVM are used to perform fault classification on selected features. The accuracy of the models depended on the type of UAV analyzed and is between 90.53 % (Quadcopter) and 100 % (for helicopter and duocopter) [13].

A. Altinors, F. Yol, and O. Yaman, used data-driven methods to find propeller eccentricity, and bearing failures in BLDC motor components of UAVs. To find the best machine learning method in predicting defects, the performances of several models such as Decision Trees (DT), SVM and KNN were compared. The models were trained using sound signals obtained by checking three different 1400 KV, 2200 KV and 2700 KV UAV motors. The features were extracted within the time domain. Model accuracy was about 99 % [1].

A. Benini, F. Ferracuti, A. Monteriù, and S. Radensleben suggested a data-based method to find the failures of the propellers of the UAVs. Signals from acceleration sensors were used and features were extracted within the time and frequency domain. The data was obtained by monitoring the flight. Model accuracy was greater than 95 % [19].

H. A. Hussain, A. N. Hussain and W. R. Abed used Artificial Neural Network (ANN) to identify BLDC motor faults such as stator winding faults, control circuit switch's fault and bearing faults. Data from an acceleration sensor as well as various electrical parameters of BLDC motors were used to train the model. The experimental results showed that the model has an accuracy of 99 % [20].

To classify various kinds of bearing defects in electric motors, C. Abdelkrim, M. Mohamed Salah, N. Boutasseta, and L. Boulanouar use the Adaptive Neuro Fuzzy Inference System (ANFIS). Training data was obtained by checking the vibration of the bearings at different rotational speeds of the motor shaft and the features were extracted within the time domain using statistical methods. The accuracy of the model for all training and testing data was about 97 % [14].

M. Khanjani and M. Ezoji used thermal imaging to detect failures of three-phase electric motors. The features were extracted using the Convolutional Neural Network (CNN), then the training data was grouped using K-Means in the cold and hot clusters. For the classification of defects, the SVM classifier algorithm can detect 100 % of the faults of the induction motor [21].

Z. Fu, X. Liu and J. Liu used the Probabilistic Neural Network (PNN) to detect faults in the windings of BLDC's motors. The training data were obtained by checking the electrical current through windings and the features were extracted in the time-frequency domain using Wavelet Transform (WT). The accuracy of the model is 100 % [5].

J.-Y. Lee, W.-T. Lee, S.-H Ko, and H.-S. Oh propose a method for diagnosing BLDC motor failures within UAVs, using a model-based method that involves the elaboration of equations of stationary state conditions. The method can classify propeller failures, stator failures, rotor failures and others. The accuracy of the method was 99 % [2].

A. Bondyra, P. Gąsior, S. Gardecki, and A. Kasiński uses a data-driven method that has a Random Forest (RF) machine learning algorithm. To find BLDC motor propeller failures, Signals from acceleration sensors were used to train the model. Features were extracted within the frequency domain using Fast Fourier Transform (FFT). The method could detect 95 % of engine faults [22].

S. Ai, W. Shang, J. Song and G. Horses used a data-driven method to find BLDC motor defects. They used GcForest as the machine learning algorithm. Signals from acceleration sensors (MPU6050) were used to train the model and the features were extracted within the time-frequency domain using Wavelet Packet Transform (WPT). The accuracy of the model was 99 % [23].

O. Jahanian and H. Koten used 11 regression models based on machine learning techniques to predict the start of combustion in homogeneous charge compression ignition engines fueled with methane The data used for this study came from virtual engines which used the detailed chemical kinetics of methane combustion. From the experimental results obtained, the authors applying machine learning techniques improve the prediction from 89,3 % to 98,4 % [24].

W. Shang, X. Liu, X. Wang and X. Wang make a model for fatigue life prediction for SiC and Al materials based on path planning algorithm considering residual stress. The modified Paris model was used to estimate the fatigue life [25].

### 1.2 Structure of the work

In this paper, the authors have conducted a new study in which they use a data-driven method to find the defects of BLDC electric motors within the UAVs. The method uses data from vibration, temperature, electrical current and voltage sensors to classify defects in the BLDC electric motors used in the UAVs. Identification of classes such as, Healthy, Chipped propeller, Eccentric shaft and ESC fault were studied. To maximize the performance of the method, the results of several machine learning algorithms in classifying the four classes were compared.

The work is organized as follows. Section I presents the importance of the methods for finding BLDC motor faults in the UAV, as well as the causes from which these faults can occur. They are also presented in this section and the main concerns of researchers in the literature on finding methods to find defects in electric motors. Section II presents the proposed method as well as the machine learning algorithms used to classify the defects of BLDC motors. To find the best algorithm that can classify BLDC motor faults, the performance of several machine learning algorithms such as KNN, SVM, and Bayesian Network (BN) was compared. In Section III, the experimental stand from which the experimental data originated is presented. Section IV the experimental setup and the data obtained from checking the operation of the BLDC motor, which were used to train the machine learning models. Section V presents the experimental results obtained. The conclusions of this study are presented in Section VI.

## 2 Methodology for identifying the types of defects of BLDC motors

To predict defects and the Remaining Useful Life (RUL) of machines with rotating components, researchers used three methods: model-based, data-driven and hybrid. Model-based approaches involved making a model that shapes the degradation process. Data-driven approaches required an ample collection of data from the sensors. These sensors captured both normal and abnormal behavior. Hybrid approaches used both methods [8].

To build a suitable degradation model, the model-based methods require advanced knowledge of the physical and chemical processes taking place.

Storing and manipulating large data is commonly used on the Internet of Things (IoT) era. Thus, extracting information from data favored data-driven methods. Data-driven methods use machine learning to estimate RUL and diagnose defects and they have a much better generalization capacity [8,23].

In this article, a data-driven method was used to find faults in BLDC motors in the UAVs, which used historical data from monitoring under the influence of different classes of faults.

The historical data was obtained by using the sensors for measuring, the vibration, temperature, electrical current and voltage, when the BLDC motor is working under the influence of classes such as, Healthy, Chipped propeller, Eccentric shaft, and ESC fault, which represent the most common defects encountered in the UAVs. Parameters such as temperature, vibration, electrical current and voltage can express the health of the motor. When the motor fails, these parameters will change. Thus, a fast method that can work in real time has been developed.

**Fig. 1:**
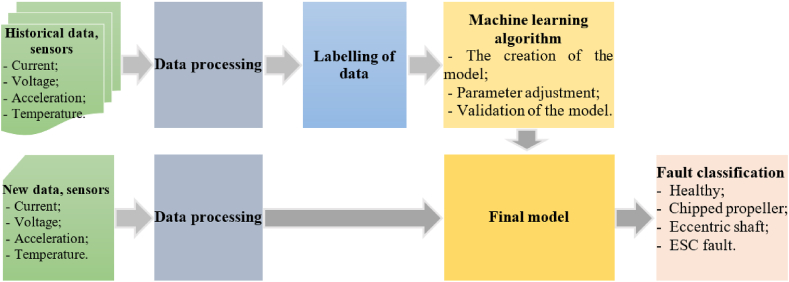
Diagram of the process used classification of defects of BLDC motors.

The diagram of the process used to name defects in real time is shown in Fig. 1.

The historical data is preprocessed and then the defective features are extracted in the data processing step, and finally labeled. They contain information about the analyzed defects.

Once the model has been created, it can be used to classify defects. Thus, the real-time data from the sensors are preprocessed and then the features of the defects are extracted, and at the end they are applied to the model to classify defects.

Both historical and new data must be preprocessed during the data processing phase. Preprocessing involves the following operations steps: noise extraction, elimination of outlier values, completion of missing values and normalization. The normalization of data *X*_*new*_ is given by (1) [1].

(1) X_(new)=(X_(old)–X_(min))/(X_(max)–X_(min))

Where: *X*_*old*_ is an old value before normalization; *X*_min_ is the value of minimum and *X*_max_ is maximum in which the data will be normalized.

In this step the features of the defects are also extracted.

Feature extraction is the process of revealing hidden information in the input signal that helps improve the model's performance to classify new data. Also, through this process the data is reduced, keeping the essential information which helps to reduce the number of resources needed to analyze an input signal. Features of defects of rotary machines can be extracted in: time domain, frequency domain and time-frequency domain [26].

The extraction of features in the time domain is often used to find defects and uses statistical indicators based on time, such as: Media, Standard Deviation, Kurtosis, Skewness, Root Mean Square (RMS), Etc. Frequency domain feature extraction methods are also used. In the frequency domain, FFT is applied to raw data to extract the power spectrum and reveal information about the features frequencies of the signal. The main disadvantage of this method of analysis, of the signals in the frequency domain, is represented by the difficulty in selecting the frequency band in which the symptoms of defects are found. Analysis in the time-frequency domain allows us to discover useful information for both stationary and non-stationary signals. Techniques of extracting the features of the time-frequency domain include: Short-time Fourier Transform (STFT), Wavelet transform, etc. [14,27,28].

In this article, the features were extracted in the time domain using time-based statistical indicators such as, Mean, Standard Deviation, Kurtosis, Skewness, RMS. The data used to train the model was purchased from the experimental stand used in this work and contains information (from sensors) about the functioning of the motor under the influence of each analyzed defect.

Finally, the training data was applied to the machine learning algorithm to create the model used to classify new data.

The defect classification problem has the following classes: Healthy, Chipped propeller, Eccentric shaft, and ESC fault (faulty FDD8896 MOSFET transistor). If a classification problem has more than two results, then multi-class classifiers are used.

The performances of algorithms such as KNN, SVM, and BN were compared, with the aim of finding the algorithm that has the best performance in classifying the faults of BLDC electric motors.

### 2.1 Using KNN to classify the fault types of BLDC motors

KNN is a nonparametrically supervised machine learning algorithm, often used in both classification and regression problems. However, it is also used for classification problems. In matters of classification the result belongs to the class [29,30].

A new instance is classified by the majority vote of its neighbors *k*, so the instance will be assigned to the class most found in the case of its neighbors *k*. Usually *k* is an integer, a small number. If *k = 1*, then the new instance will be assigned to the class to the nearest neighbor. The distance between the sample instance and its neighbor can be calculated using: Euclidean distance, Manhattan distance, Chebyshev distance and Hamming distance. The Euclidean distance between a test instance *x'* and each instance in the formation dataset *x*, is (2) [31], [32].

(2) d(x,x’)=sqrt(∑(x–x’))

Distances are ordered in ascending order, then only the nearest *k* instances will be considered. The choice of the best value for *k* is made by analyzing the data. If a small value is chosen for *k*, the quality of the classification is affected by the noise, and a value too high for *k* will reduce the influence of the noise on the classification, but will make the areas separating the classes less distinct [33]. In the *Python* language, KNN can be implemented using the *Scikit-learn* module.

### 2.2 Using SVM to classify BLDC motor fault types

SVM are machine learning techniques often used in both classification and regression problems, being more used in classification problems. SVMs can be used in the classification of linear, nonlinear, and multiclass data. The use of SVMs has a number of advantages such as: they are effective with large data sizes; the memory is managed efficiently because it uses a subset of training points in the decision function called support vectors. In *Python* language, SVM can be implemented using the *Scikit-learn* module [[34], [35], [36], [37], [38]].

To classify linear data, the SVM algorithm decides the hyperplane that can segregate *n*-dimensional space into classes so that we can correctly classify new data points. The size of the hyperplane depends on the number of features. If we have two input features, then the hyperplane is a line and if we have three features then the hyperplane becomes a 2D plane. The hyperplane shall be chosen in such a way that the distance between it to the nearest points of the separate classes is maximum. The data points that are closest to the hyperplane are called support vectors. The optimization problem in the case of the linear SVM classifier consists, in finding the parameters *w*, *ξ* and *b* so that the edge is maximum for all instances *(x*_*i*_*, y*_*i*_*)*, so we have (3) [35], [36]:

$$\min_{w,\xi ,b}\left(\left({\left\|w\right\|}^{2}\right)/2\right)+C\;\sum_{\text{xi}\;\epsilon \;X}\xi_{i}$$

(3) $$With\;the\;constraints:\xi_{i}\geq 0\;\text{and}\;y_{i}\;\left[w\;ѱ\;\left(x_{i}\right)+b\right]\geq 1\;-\;\xi_{i}$$

Where: *ξ*_*i*_ is the slack variable, *C ≥ 0* is the penalty factor, *X* is the input vector, *‖w‖*^*2*^ is the second-order norm of the weight vector and function *ψ* is used to map the input data *X* to a higher-dimensional space. The previous form of the optimization problem is adapted for data that is not perfectly separated by a hyperplane. The problem is solved with the help of Lagrange multipliers [35,36].

To classify nonlinear data, the SVM maps the input data to a higher dimensional space *n* + *1* to make it linearly separable. This mapping is nonlinear and is implemented using kernel functions. Within this method it is difficult to choose the right kernel function [39,40].

### 2.3 Using the BN classifier to classify the fault types of BLDC motors

The BN classifier is a probabilistic classifier based on Bayes' theorem that assumes that each feature depends only on the class. It assumes that each feature is independent and does not interact with each other [[41], [42], [43]].

Starting from a training dataset for the target function and a new training instance (*X*_*1*_*,X*_*2*_*, …,X*_*m*_*, m* is the total number of instances), for which we must predict the *C*_*j*_ class to which the instance belongs, through the Bayesian approach to the new instance we will assign it the most likely class. The most likely target *C*_*P*_ can be expressed by (4) [41], [44]:

(4) $$C_{P}=\left(\mathrm{argmax}\right)P\left(C_{j}\;\right)\;\prod_{i=1}^{m}\;P\left(X_{i}│C_{j}\right)$$

Where: *P(C*_*j*_*)* is class probability and *P(X*_*i*_*│C*_*j*_*)* is the probability of instance with number *i*, *X*_*i*_ to be in class with number *j, C*_*j*_. So, we must discover the value of the *C*_*j*_ class for which the value of the product is maximum for the new instance. *P(C*_*j*_*)*, *P(X*_*i*_*│C*_*j*_*)* are estimated, based on the number of occurrences in the training data [41].

Despite its simplicity, the naïve Bayesian classifier produces exceptionally good results and is intensively used because it often goes beyond more sophisticated classification methods. It is computationally fast, simple to implement, and works well on high-dimensional datasets.

### 2.4 Performance analysis of classifiers

Measuring the performance of a multiclass classifier is like measuring the performance of a binary classifier. The main indicators used to measure the performance of classifiers are: Accuracy (*ACC*), Precision (*PR*_*class*__*j*_), Recall (*Recall*_*class*__*j*_) and F1 Score (*F1 Score*_*class j*_). Precision, F1 Score and Recall are calculated separately for each class (5) [32], [45].

Precision explains how many of the correctly predicted cases turned out to be positive. Recall specifies how many of the actual positive cases were correctly predicted. The F1 score is the harmonic mean of accuracy and sensitivity. ACC is the probability that the prediction of the model will be correct [32,45].

$${\mathrm{PR}}_{class\;j}=\left({TP}_{class\;j}\right)/\left({TP}_{class\;j}+{FP}_{class\;j}\right)$$

$${Recall}_{class\;j}=\left({TP}_{class\;j}\right)/\left({TP}_{class\;j}+{FN}_{class\;j}\right)$$

$$F1\;{Score}_{class\;j}=\left(2\;{\mathrm{PR}}_{class\;j}\;{Recall}_{class\;j}\right)/\left({\mathrm{PR}}_{class\;j}+{Recall}_{class\;j}\right)$$

(5) ACC=(TP+TN)/(TP+TN+FP+FN)

Where: TP is the truth positive rate; FP is the false positive rate; TN is the true negative rate; FN is the false negative rate; *TP*_*class j*_ is the truth positive rate for class *j*; *FP*_*class j*_ is the false positive rate for class *j*; *FN*_*class j*_ is the false negative rate for class *j* [32,45,46].

To express the performance of all classes with a single value, it is used: *PR*_*macro avg*,_ *Recall*_*macro*_ _*avg*_ and *F1score*_*macro avg*_. Where: *PR*_*macro avg*_ is the macro average precision and represents the arithmetic mean of all the *PR*_*class j*_ values for the *j* classes, *Recall*_*macro avg*_ is the macro average recall and represents arithmetic mean of all *Recall*_*class j*_ value for the *j* classes and *F1score*_*macro avg*_ is calculated as arithmetic mean of all *F1 Score*_*class j*_ value for the *j* classes [47].

## 3 The experimental stand used to extract historical data

**Fig. 2:**
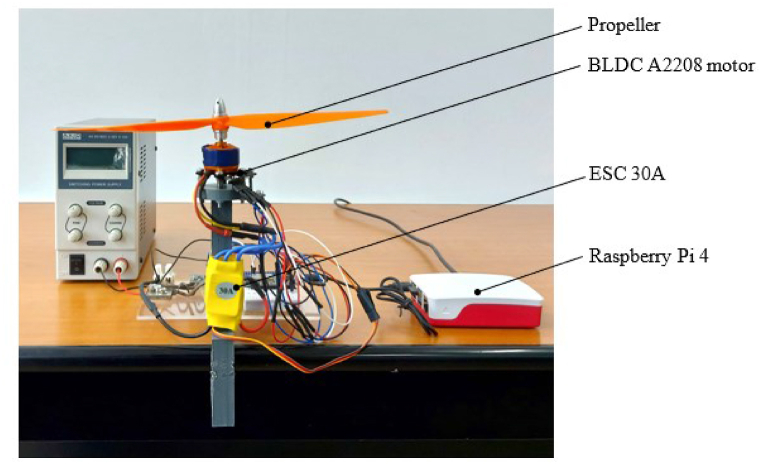
Experimental stand, front view.

**Fig. 3:**
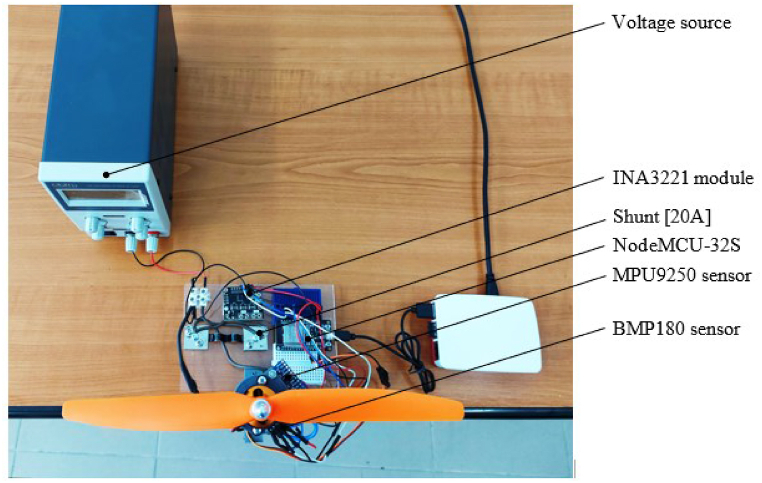
Experimental stand, top view.

The experimental stand used to check BLDC motors allows temperature, vibration, electrical current and voltage monitoring through sensors to find its defects. The experimental stand consists of the BLDC motor as shown in Fig. 2, whose speed is controlled via the ESC as illustrated in Fig. 2 by the NodeMCU-32S development board as shown in Fig. 3, which includes the ESP32 SOC module. The propeller, as proved in Fig. 2, stands in solidarity with the shaft of the BLDC motor.

During the operation of the BLDC motor, to find its faults, the MPU9250 sensor checks the acceleration on the three axes and the BMP180 sensor checks the temperature, as illustrated in Fig. 3.

The supply voltage of the ESC and the electrical current that closes through the motor were checked via the INA3221 module as shown in Fig. 3. Since the INA3221 module supports an electrical current of 3.5 [A] and the maximum electrical current consumed by the motor is 7.5 [A], a shunt was used to extend the measuring range to 20 [A] as shown in Fig. 3. Data from the acceleration sensor, temperature sensor and the module used to check electrical parameters are transmitted via the I2C protocol to the NodeMCU development board that transmits it further to a Raspberry Pi 4 as illustrated in Fig. 2 for their storage. The supply of the stand is made through the voltage source as shown in Fig. 3.

In this study, BLDC motors model A2208 were used. The datasheet of the motors is given in Table 1.

**Table 1:** Datasheet of motors A2208.

Parameter	Value	Unit
KV(RPM/Volt)	1100	[kV]
Maximum electrical current	8/60	[A/s]
Impedance	225	[mΩ]

Usually in UAV motors the most common failures include rotor-shaft failures, bearing failures, propeller failures, ESC failures as well as winding failures on the stator.

For this study, the following faults were created, chipped propeller, eccentric shaft, and ESC fault, commonly found in BLDC motors in UAVs.

**Fig. 4:**
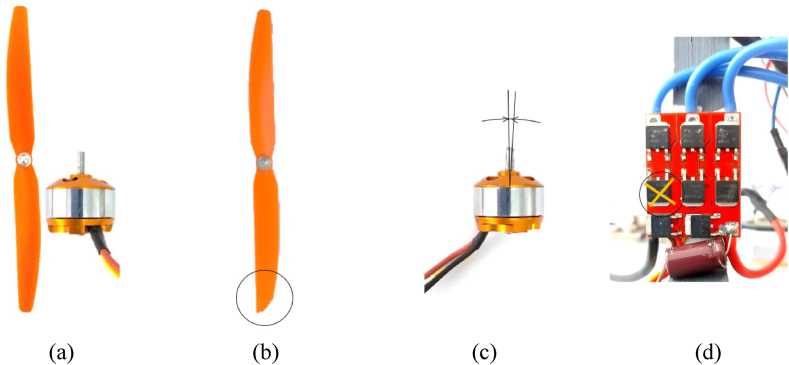
Classes analyzed. (a) *C1* - Healthy class. (b) *C2* - Chipped propeller class. (c) *C3* - Eccentric shaft class. (d) *C4* - ESC fault class (faulty FDD8896 MOSFET transistor).

The analyzed classes in this study are presented in Fig. 4. The dataset was divided into four classes: *C1* - Healthy class, *C2* - Chipped propeller class, *C3* - Eccentric shaft class, *C4* - ESC fault class (faulty FDD8896 MOSFET transistor). In Fig. 4(a) is shown the class *C1*, in which the motor works without defects, in Fig. 4(b) is given the class *C2¸* where the motor works with the chipped propeller defect, in Fig. 4(c) is presented the class *C3*, where the motor works in this class under the influence of the eccentric shaft defect, and in Fig. 4(d) is depicted the class *C4*, in which the motor works with a defective MOSFET transistor, respectively.

Each class was implemented and analyzed using the experimental stand, thus, experimental data holding information about each analyzed class were generated. During monitoring, the motor runs at different speeds.

## 4 Experimental setup

**Fig. 5:**
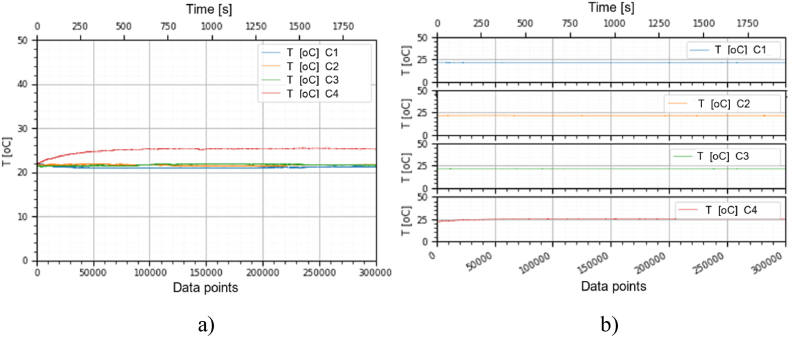
Variation *T* [°C] for classes analyzed *Cj (j = 1, 2, 3, 4)* at rotational speed of 1000 [RPM] during the 300000 data points acquired. (a) Results are presented in the same graph. (b) Results are shown in different graphs.

**Fig. 6:**
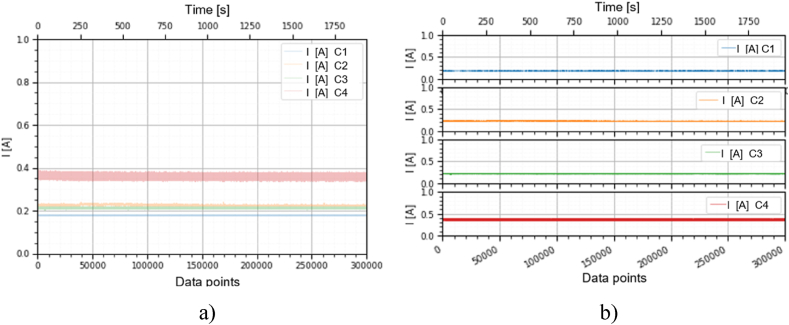
Variation *I* [A] for the classes analyzed *Cj (j = 1, 2, 3, 4****)*** at the rotational speed of 1000 [RPM] during the 300000 data points acquired. (a) Results are presented in the same graph. (b) Results are shown in different graphs.

**Fig. 7:**
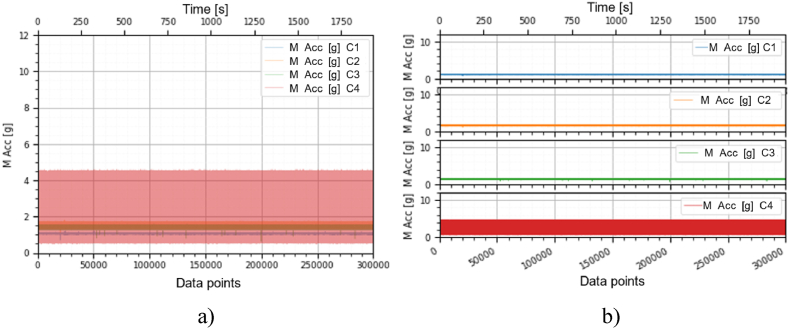
Variation *M Acc* [g] for the classes analyzed *Cj (j = 1, 2, 3, 4)* at the rotational speed of 1000 [RPM] during the 300000 data points acquired. (a) Results are presented in the same graph. (b) Results are shown in different graphs.

**Fig. 8:**
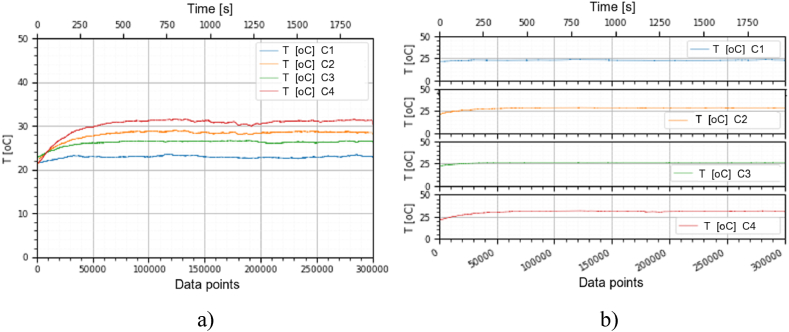
Variation *T* [°C] for the classes analyzed *Cj (j = 1, 2, 3, 4)* at rotational speed of 2000 [RPM] during the 300000 data points acquired. (a) Results are presented in the same graph. (b) Results are shown in different graphs.

**Fig. 9:**
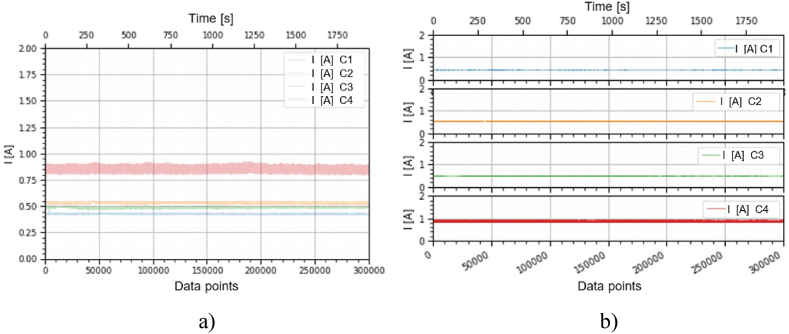
Variation *I* [A] for the classes analyzed *Cj (j = 1, 2, 3, 4)* at the rotational speed of 2000 [RPM] during the 300000 data points acquired. (a) Results are presented in the same graph. (b) Results are shown in different graphs.

**Fig. 10:**
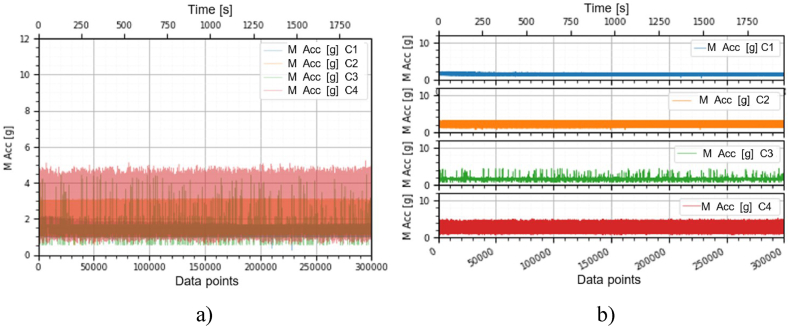
Variation *M Acc* [g] for the classes analyzed *Cj (j = 1, 2, 3, 4)* at rotational speed of 2000 [RPM] during the 300000 data points acquired. (a) Results are presented in the same graph. (b) Results are shown in different graphs.

**Fig. 11:**
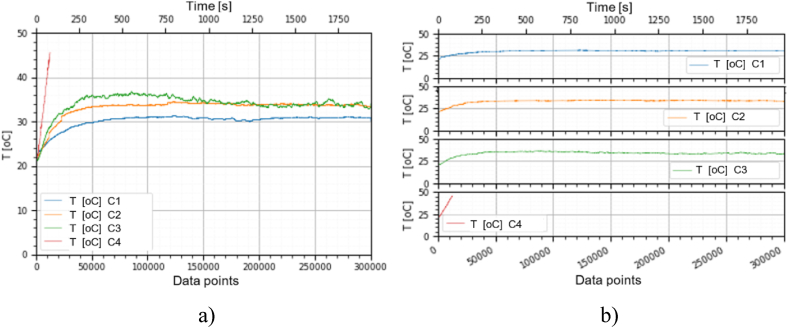
Variation *T* [°C] for the classes analyzed *Cj (j = 1, 2, 3, 4)* at rotational speed of 4000 [RPM] during the 300000 data points acquired. (a) Results are presented in the same graph. (b) Results are shown in different graphs.

**Fig. 12:**
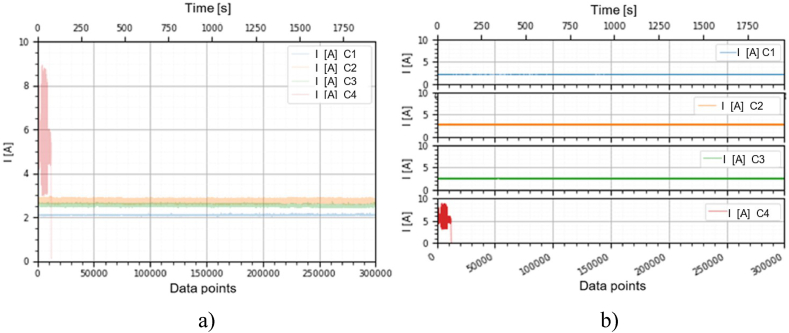
Variation *I* [A] for the classes analyzed *Cj (j = 1, 2, 3, 4)* at rotational speed of 4000 [RPM] during the 300000 data points acquired. (a) Results are presented in the same graph. (b) Results are shown in different graphs.

**Fig. 13:**
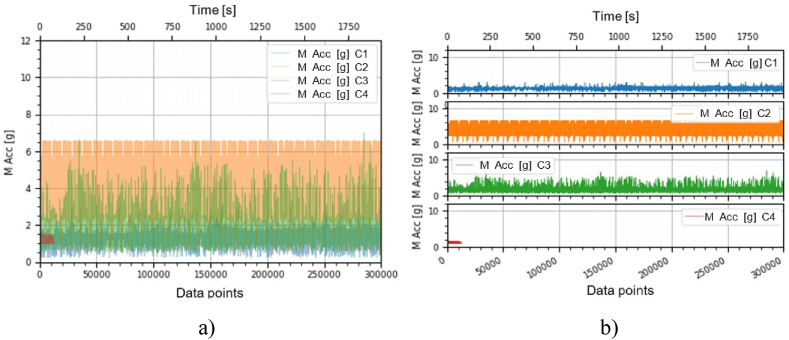
Variation *M Acc* [g] for classes analyzed *Cj (j = 1, 2, 3, 4)* at rotational speed of 4000 [RPM] over the 300000 data points acquired. (a) Results are presented in the same graph. (b) Results are shown in different graphs.

This section holds the data obtained from checking the functioning of the BLDC motors, which were used to train the machine learning models. A representation of the variation, temperature (*T* [°C]), electrical current intensity (*I* [mA]), acceleration magnitude (*M Acc* [g]) for the analyzed classes, at the speeds of, 1000 [RPM], 2000 [RPM] and 4000 [RPM] is illustrated in Fig. 5, Fig. 6, Fig. 7, Fig. 8, Fig. 9, Fig. 10, Fig. 11, Fig. 12, Fig. 13, where the caption part (a) represent the results displayed in the same graph and the caption part (b) represent the results displayed in different graphs, in which the notations were used: *C1* - Healthy class; *C2* - Eccentric shaft class; *C3* - Chipped propeller class; *C4* - ESC fault class (faulty FDD8896 MOSFET transistor).

From the graphs in Fig. 5, Fig. 6, Fig. 7, Fig. 8, Fig. 9, Fig. 10, which indicates the variations in the parameters *T* [°C], *I* [A] and *M Acc* [g] for the classes analyzed *Cj (j = 1, 2, 3, 4)* at the speed of 1000 [RPM] and 2000 [RPM], it is found that *M Acc* [g] has a large amplitude variation for the ESC fault class in the rest, not very large differences between the parameters are found.

For the ESC fault class, presented in Figs. 11–13 at a speed of 4000 [RPM], the ESC interrupted the operation of the motor after about 80 [s], entering protection mode due to the amplitude and high variation of the motor supply electrical current.

From the graphs of variation of the parameters *T* [°C], *I* [mA], *M Acc* [g], in relation to the points purchased, at the motor shaft speeds of, 1000 [RPM], 2000 [RPM] and 4000 [RPM] depicted in Figs. 5–13, it can be ascertained:

- *T* [°C], *I* [mA], *M Acc* [g], have the highest amplitudes for the ESC fault (faulty FDD8896 MOSFET transistor) class, unlike the other classes. The motor has an extremely high energy consumption for this class and runs noisily. To obtain the ordered revs, the motor consumes much more, double that of the healthy class.
- *T* [°C], *I* [mA], *M Acc* [g], have the lowest amplitudes for the healthy class. The motor vibrates extraordinarily little, and the energy consumption is minimal.
- the values of the parameters *T* [°C], *I* [mA], *M Acc* [g] vary depending on the rotational speed of the rotor shaft. They increase with increasing speed.
- each analyzed class has a different variation of the parameters *T* [°C], *I* [mA], *M Acc.* [g], thus creating a unique imprint for each class.
- faulty operation causes increased noise and energy consumption.

## 5 Results and discussions

The proposed method was implemented using *Python 3.6* with *Tensorflow* and *Pandas* packages. Experimental results were obtained using a PC with an i7-6500U CPU @ 2.50 GHz processor and 12.0 GB of RAM.

Three BLDC motors, three propellers and two ESC were used to generate the experimental results. The model training dataset has information about each class. Each class has data in which the variation of the parameters of *T* [°C], *I* [mA], *M Acc* [g] was monitored in relation to the variation of the rotational speed of the motor shaft in the range of 0–4000 [RPM]. The variation of the parameters coming from sensors *T* [°C], *I* [mA], *M Acc* [g] is different for each individual class. Classes create unique fingerprints on data.

For the three classifiers used, the indicators were calculated: *ACC*, *PR*_*macro*_ _*avg*_, *Recall*_*macro*_ _*avg*_ and *F1*_*macro avg*_. As seen in Table 2, the best performance in the classification of BLDC motor defects was achieved by the SVM classifier model. The worst performance to classify BLDC motor defects was obtained by the BN classifiers. The performances of the KNN classifiers are similar to the SVM classifier.

**Table 2:** Performance of the algorithms used to find the faults of BLDC motors.

Replace method			
Replace method Performance indicator	SVM	KNN	BN
*ACC*	0.96	0.95	0.91
*PR*_*macro*__*avg*_	0.96	0.94	0.90
*Recall*_*macro*__*avg*_	0.97	0.95	0.91
*F1*_*macro*__*avg*_	0.96	0.95	0.91

The features were extracted using time-domain statistical indicators such as, Mean, Standard Deviation, Kurtosis, Skewness, RMS.

The performances of the classifiers can be improved if more datasets are used for training in which we have, more propellers with different chip shapes but also BLDC motors with different angles of bent axes. The performance of the classifiers can also be improved by increasing the number of monitored parameters of the BLDC motor.

According to results obtained in *Python 3.6*, class 4 was the most correctly classified class. This is because the training data contains very different sensor values for class 4 compared to sensor values for the other classes, therefore class 4 cannot be easily confused with other classes, Fig. 5, Fig. 6, Fig. 7, Fig. 8, Fig. 9, Fig. 10, Fig. 11, Fig. 12, Fig. 13.

The training was done using *K-fold* cross-validation with *k = 5*. The SVM algorithm is much faster compared to KNN and BN algorithms. The slowest algorithm is BN.

## 6 Conclusions

In this paper, the authors present a novel approach to fault classification of BLDC motors from UAVs, using machine learning. In order to maximize the defect classification performance, the performances of the SVM, KNN and BN classifiers were compared in classifying defects. The performances of the SVM classifier are higher compared to the other classifiers.

The training data set of the model contains the data from monitoring the operation of BLDC motors under the influence of classes such as: Healthy class, Chipped propeller class, Eccentric shaft class, ESC fault (faulty FDD8896 MOSFET transistor) class. The following were used to generate the training data in this study: a good propeller, a chipped propeller, a good BLDC motor, an eccentric shaft BLDC motor, a good ESC, an ESC with faulty FDD8896 MOSFET transistor. If the defects of the studied components have different characteristics such as the propeller chip has a different shape of the shaft is bent at a different angle, then the accuracy of the model to identify the defects will decrease. The generalizability of the model will increase if the training data contains information from a larger number of faulty propellers and BLDC motors whose axes are eccentric.

The features of each class are different, thus creating a unique fingerprint on the data acquired from the sensors. This can be seen Fig. 5, Fig. 6, Fig. 7, Fig. 8, Fig. 9, Fig. 10, Fig. 11, Fig. 12, Fig. 13 where we have the variation of parameters T [°C], I [mA], M Acc [g] for each analyzed class, C1, C2, C3 and C4.

From the resulting experimental data, it is found that when the BLDC motor operates under the influence of the ESC fault (faulty FDD8896 MOSFET transistor) class, the motor operates noisy, the power consumption is high, and the temperature is high, unlike the operation under the influence of the other classes. Finally, prolonged operation below this class leads to the loss of insulation of the winding on the stator, which leads to the occurrence of functional failure.

As a result of this study, it was found that the proposed model presents more realistic predictions than those of other models, having a greater generalization ability. It can be implemented within a PdM system of a UAV, to find faults in BLDC motors.

## Funding

This research of Andrioaia Dragos-Alexandru was supported by the project "PROINVENT", Contract no. 62487/03.06.2022—POCU/993/6/13—Code 153299, financed by The Human Capital Operational Programme 2014–2020 (POCU) Romania. The “Vasile Alecsandri” University of Bacau funded the APC.

## Data availability statement

All data required to support this study is already mentioned in the manuscript.

## CRediT authorship contribution statement

**Dragos Alexandru Andrioaia:** Software, Data curation, Conceptualization. **Vasile Gheorghita Gaitan:** Supervision.

## Declaration of competing interest

The authors declare that they have no known competing financial interests or personal relationships that could have appeared to influence the work reported in this paper.
